# Examining trends in cardiovascular disease mortality across Europe: how does the introduction of a new European Standard Population affect the description of the relative burden of cardiovascular disease?

**DOI:** 10.1186/s12963-019-0187-7

**Published:** 2019-05-30

**Authors:** Shiva Tadayon, Kremlin Wickramasinghe, Nick Townsend

**Affiliations:** 1 Centre on Population Approaches for Non-Communicable Disease Prevention, Nuffield Department of Population Health, University of Oxford, Oxford, UK; 2 UCLA Comprehensive Vascular Neurology Program, Department of Neurology, University of California at Los Angeles, 710 Westwood Plaza, Los Angeles, CA 90095-17693 USA; 3 World Health Organization European Office for Prevention and Control of Noncommunicable Diseases (NCD Office), Moscow, Russian Federation; 4 Department for Health, University of Bath, Bath, BA2 7AY UK

**Keywords:** Cardiovascular disease, Epidemiology, European Standard Population, Mortality

## Abstract

**Background:**

Some mortality statistics are misleading when comparing between countries due to varying age distributions in their populations. In order to adjust for these differences, age-standardised mortality rates (ASMRs) are often produced. ASMRs allow for comparisons between countries as if both had the same standardised population. We examined whether the updating of the standard population for Europe affected the description of the relative burden between countries in cardiovascular disease (CVD) mortality across the continent.

**Methods:**

Mortality and population data were obtained from the World Health Organization (WHO) mortality database. ASMRs were calculated using the direct method and two European Standard Populations (ESP): 1976 ESP and 2013 ESP. We investigated differences in ASMR76 (calculated using 1976 ESP) and ASMR13 (calculated using 2013 ESP), changes in rankings of countries between the two ASMRs and differences in trends in CVD mortality in each country for the two ASMRs.

**Results:**

CVD rates calculated using the 1976 ESP were on average half the size of rates calculated using the 2013 ESP. Spearman’s rank coefficient showed that the ranks of countries by ASMRs calculated using the two ESPs were different for both sexes. Joinpoint analyses showed no difference in the direction of trend between ASMR76 and ASMR13 although differences in the magnitude of the change were found in some countries.

**Conclusion:**

ASMRs are commonly used in studying the epidemiology of a disease. It is crucial that policy makers understand the effect of changes in standard populations on these rates. This includes how populations with different age distributions compare to each other. Similar effects may be seen in other diseases that are also more prevalent in older age groups, such as cancer and dementia.

## Introduction

Despite large decreases in cardiovascular disease (CVD) mortality within Europe over the last four decades [1–3], CVD remains the leading cause of mortality in the continent [4]. More than 4 million deaths are attributed to CVD in Europe annually, accounting for 46% of all deaths, with the number of CVD deaths higher in women (2.2 million) than men (1.8 million) [2].

Despite decreasing trends in mortality from CVD found in most European countries, there is great variation in the extent of this decline between countries. For example, 10-year decreases in CVD age-standardised mortality rates (ASMRs) ranged from 1.3% and 6.3% for men and women respectively in Kyrgyzstan to 56.5% and 65.6% respectively in Kazakhstan [2]. It is no surprise that large inequalities remain across Europe in the relative and absolute burden of CVD [5, 6] with CVD-related mortality generally higher in Central and Eastern Europe [1, 2, 5, 7].

Comparing between countries and sexes can be problematic due to differences in population structure. Since CVD is more common in older age groups, countries or sexes with a greater proportion of older individuals may be expected to suffer a higher proportion of CVD deaths [8]. ASMRs are used to account for some differences in age and population structure and have been used to compare between European countries in the burden of many CVD outcomes, including mortality. ASMRs are calculated by applying age-specific mortality rates for different countries to the same standard population. Rates can differ depending on which standard population is used.

The European Standard Population (ESP) is recommended for the calculation of ASMRs in Europe. However, Eurostat, the statistical office of the European Union, updated the 1976 ESP in 2013 to reflect better the current European population structure that had changed from 1976 due to an increase in life expectancy [9]. The 2013 ESP has greater weighting on older age groups and has an upper limit disaggregated to include age groups of 90 years and older, compared to the 1976 ESP upper age limit of 85 and over [10].

The introduction of the 2013 ESP has been shown to lead to ASMRs in CVD about twice as large as those calculated using the 1976 ESP [2]. This increase may not be the same for all countries and sexes as there is a greater emphasis on older age groups in the 2013 ESP. Those with a greater proportion of older age groups liable to show relatively larger increases. This may change how we view inequalities in CVD mortality within Europe. We know that the change in standard population will change the ASMRs calculated; if this change is uniform across all countries, then the description and presentation of inequalities across Europe are unaffected. However, if this change in standard population leads to heterogeneous changes in ASMRs between countries, this may change the narrative around inequalities in CVD mortality across the continent.

In this study, we aimed to examine how updating the ESP changed the CVD ASMRs calculated for European countries and whether the extent of any relative difference varied by country. We calculated CVD ASMRs for each country and both sexes using both European Standard Populations, ESP13 and ESP76. We examined changes to ASMRs due to the change in ESP and examined changes in the trends in CVD mortality expressed through ASMRs calculated using the 1976 and 2013 ESPs. Joinpoint analysis was used to analyse trends in CVD ASMRs for both ESPs in each country over time and by sex.

## Methods

Data on cause-specific numbers of deaths and population numbers, by sex and in 5-year age groups (up to 85 and over) for European countries were extracted from the WHO global mortality database. The WHO database collates data reported by national authorities based on their civil registration systems and contains data for 51 of 53 European countries. Data for each country were extracted and analysed for the years of 1965 to 2014, where available. Countries were excluded if both population and mortality data for that year were not available (Andorra and Monaco).

Cardiovascular disease as a cause of death was defined according to the following International Classification of Disease codes: ICD-10 (International Classification of Diseases, tenth revision) codes I00-I99; ICD-9 codes 390-459, ICD-8 codes A081-A088 and ICD-7 codes A080-A086. Age and sex-specific mortality rates were calculated and the direct method was used to calculate age-standardised mortality rates for both the ESP76 (ASMR76) and ESP13 (ASMR13). These rates are presented for the ‘most recent year’, which are related to the most recent data for which both mortality and population data were available.

Countries were ranked by CVD ASMRs with differences in rankings between ASMR76 and ASMR13 examined through Spearman’s rank test. All statistical analyses were performed using STATA (version 14.0)**.**

Trends in ASMRs for both ESPs were calculated. Joinpoint Trend Analysis Software (version 4.3.1.0) was used to perform joinpoint regressions to identify periods with statistically distinct log-linear trends in death rates from CVD over time within each age group, by sex and country. We used joinpoint to calculate the average annual percentage change (AAPC) over the entire period of available data and the annual percentage change (APC) for individual trend segments. Segments are identified by inflexion points (‘joinpoints’) at which there is a significant change in trends, using a series of permutation tests, with Bonferroni adjustment for multiple comparisons. The changes in trend may include any change in intensity, but could include a change in direction. We used a two-sided significance level set at *P* < 0.05 for all tests. Significant joinponts for each country by sex (maximum of 5) were determined using a log-linear model, and the annual percentage change (APC) within each segment calculated. The use of a log-linear model enables the analysis of constant percentage (rather than absolute) change in prevalence over time. Plateauing of the most recent trend for a country was defined as the trend in the most recent segments showing either a less steep decline than the preceding segment, no significant difference to zero, or an increase. Joinpoint segments were compared between ASMRs from both ESPs to examine differences in trends.

## Results

On average, 35 years of data were available for all countries. Complete data for all 49 years were only available for two countries, Malta and Austria, with 30 years or more of data available for 33 (64.7%) countries. Less than 10 years of data were available for three countries: Cyprus (9 years), San Marino (7 years) and Turkey (4 years). The number of total CVD deaths was greater in women than men in 42 out of 51 countries for the most recent available year. Furthermore, a higher percentage of total deaths that were from CVD were found in women in 48 out of 51 countries (Table 1).

**Table 1:** Overview of data availability and number of deaths, population by country and sex, most recent available year

Country	Data range (years)	Total years of data available (% of total years)*	Males		Females	
			Total population	Total no. CVD deaths (% of all deaths in that country that are from CVD)**	Total population	Total no. CVD deaths (% of all deaths in that country that are from CVD)**
Albania	1987–2004	37 (75)	1,558,376	4679 (47)	1,568,887	4212 (54)
Armenia	1981–2012	25 (51)	1,450,560	6494 (46)	1,573,567	6836 (51)
Austria	1965–2014	49 (100)	4,176,550	13,964 (37)	4,367,382	19,172 (47)
Azerbaijan	1981–2007	23 (47)	4,231,550	13,534 (51)	4,349,750	14,110 (59)
Belarus	1981–2011	26 (53)	4,403,227	35,509 (50)	5,069,945	34,678 (55)
Belgium	1965–2012	47 (96)	5,451,780	14,299 (27)	5,643,070	17,157 (31)
Bosnia and Herzegovina	1985–2011	41 (84)	1,875,931	8503 (47)	1,963,806	9887 (58)
Bulgaria	1965–2012	47 (96)	3,555,925	34,456 (61)	3,749,973	37,188 (70)
Croatia	1985–2013	29 (59)	2,053,788	10,445 (41)	2,201,901	13,787 (54)
Cyprus	2004–2012	9 (18)	420,010	1015 (35)	443,932	1004 (38)
Czech Republic	1986–2013	28 (57)	5,161,617	23,701 (24)	5,349,102	28,030 (52)
Denmark	1965–2012	49 (98)	2,771,208	6442 (25)	2,815,877	6654 (25)
Estonia	1981–2012	29 (59)	620,643	3491 (46)	708,668	4848 (62)
Finland	1965–2013	48 (98)	2,673,499	9575 (37)	2,765,485	10,079 (39)
France	1965–2011	46 (94)	30,630,778	64,659 (24)	32,593,667	74,025 (28)
Georgia	1981–2014	34 (69)	1,776,700	9664 (39)	1,950,300	10,933 (45)
Germany	1990–2013	24 (49)	39,469,105	153,309 (36)	41,176,500	201,184 (43)
Greece	1965–2012	47 (96)	5,431,556	23,438 (39)	5,661,227	26,290 (46)
Hungary	1965–2013	48 (98)	4,709,677	27,598 (45)	5,183,416	35,379 (55)
Iceland	1965–2009	45 (92)	161,548	381 (37)	157,698	348 (36)
Ireland	1965–2012	46 (94)	2,269,612	4779 (32)	2,315,795	4701 (33)
Israel	1975–2013	39 (80)	3,991,346	4819 (24)	4,068,110	5217 (25)
Italy	1965–2012	37 (76)	28,808,103	99,659 (34)	30,731,623	130,498 (41)
Kazakhstan	1981–2012	32 (65)	8,100,113	24,533 (31)	8,691,315	18,542 (29)
Kyrgyzstan	1981–2013	31 (63)	2,827,672	9017 (46)	2,892,180	8610 (57)
Latvia	1980–2012	43 (88)	930,696	6877 (50)	1,103,623	9436 (62)
Lithuania	1981–2012	32 (65)	1,376,201	9884 (48)	1,611,572	13,286 (66)
Luxembourg	1965–2013	48 (98)	271,765	523 (30)	271,595	650 (34)
Malta	1965–2014	49 (100)	213,607	577 (35)	213,814	654 (40)
Montenegro	2000–2009	10 (20)	311,262	1518 (50)	320,282	1700 (60)
Netherlands	1965–2013	48 (98)	8,320,868	18,026 (26)	8,483,577	20,437 (28)
Norway	1965–2013	48 (98)	2,551,676	5630 (28)	2,528,498	6455 (30)
Poland	1965–2013	48 (98)	18,411,126	82,518 (41)	19,620,506	94,910 (51)
Portugal	1965–2013	45 (92)	4,976,865	13,980 (26)	5,480,441	17,546 (33)
Republic of Moldova	1981–2013	31 (63)	1,711,931	9994 (50)	1,846,646	12,136 (67)
Romania	1969–2012	43 (88)	9,770,353	71,117 (53)	10,289,829	82,254 (67)
Russian Federation	1980–2011	32 (65)	66,113,269	484,763 (49)	76,847,639	589,956 (64)
San Marino	1995–2005	7 (14)	14,637	49 (42)	15,205	50 (49)
Serbia	1998–2013	11 (22)	3,488,466	24,499 (48)	3,675,666	28,859 (58)
Slovakia	1992–2010	18 (37)	2,639,896	12,856 (47)	2,791,128	15,682 (61)
Slovenia	1985–2010	26 (53)	1,014,709	3071 (33)	1,034,545	4260 (46)
Spain	1965–2013	48 (98)	22,900,000	53,487 (27)	23,700,000	63,997 (34)
Sweden	1965–2013	48 (98)	22,933,751	15,972 (37)	3,659,485	17,597 (38)
Switzerland	1965–2013	48 (98)	3,995,315	9719 (31)	4,094,044	11,793 (35)
TFYR Macedonia	1991–2010	20 (41)	1,029,724	5500 (54)	1,024,767	5566 (62)
Tajikistan	1981–2004	22 (45)	3,365,837	6691 (46)	3,344,334	6448 (53)
Turkey	2009–2013	4 (8)	38,164,870	70,476 (36)	37,889,747	71,750 (45)
Turkmenistan	1981–1998	28 (57)	2,337,600	6314 (38)	2,370,000	6847 (52)
The UK	1965–2013	48 (98)	31,532,873	79,935 (29)	32,572,781	79,860 (27)
Ukraine	1981–2012	32 (65)	20,969,728	186,857 (57)	24,443,259	249,569 (74)
Uzbekistan	1981–2005	25 (51)	13,069,360	39,235 (53)	13,097,660	39,877 (60)

*Years of available data expressed in relation to a total of 1965 to 2014

**CVD deaths expressed as a percentage of total deaths by sex, e.g. percentage of all deaths in males that were caused by CVD

CVD rates calculated using the 1976 ESP were on average half the size of rates calculated using the 2013 ESP (mean rate difference = 1.95; *P* < 0.001). The mean rate difference was 1.86 for men and 2.03 for women. ASMR13s were more than twice as large as ASMR76s in males for eight countries (Austria, Iceland, Italy, Kazakhstan, Netherlands, Norway, San Marino, Sweden, and Switzerland). In females, ASMR13s were more than twice as large as ASMR76s for more than three quarters of countries (Table 2).

**Table 2:** Rates (per 100,000) and rate difference for most recent year for all countries by ESP and sex

Country	Males			Females		
	1976 ESP rate	2013 ESP rate	Rate difference	1976 ESP rate	2013 ESP rate	Rate difference
Albania	490.7	950.7	1.94	354.8	724.5	2.04
Armenia	524.5	946.5	1.80	356.5	743.9	2.09
Austria	224.5	457.1	2.04	156.3	348.0	2.23
Azerbaijan	616.8	1078.1	1.75	488.9	944.7	1.93
Belarus	868.0	1448.0	1.67	394.0	726.9	1.84
Belgium	181.1	357.1	1.97	118.7	252.9	2.13
Bosnia and Herzegovina	474.7	918.6	1.93	385.4	805.2	2.09
Bulgaria	705.5	1299.5	1.84	469.1	959.6	2.05
Croatia	392.8	761.4	1.94	269.0	581.2	2.16
Cyprus	217.7	428.8	1.97	155.2	343.9	2.22
Czech Republic	384.7	747.6	1.94	251.0	538.2	2.14
Denmark	170.1	337.6	1.98	107.6	229.9	2.14
Estonia	501.0	920.2	1.84	269.3	572.4	2.13
Finland	250.0	480.7	1.92	136.3	295.5	2.17
France	141.0	275.2	1.95	81.0	174.1	2.15
Georgia	450.0	891.6	1.78	302.6	608.7	2.01
Germany	239.2	476.3	1.99	165.2	361.2	2.19
Greece	257.1	485.0	1.89	180.6	391.3	2.17
Hungary	494.1	921.3	1.86	310.7	646.3	2.08
Iceland	218.6	441.6	2.02	131.9	297.5	2.26
Ireland	214.0	420.5	1.97	134.9	290.2	2.15
Israel	130.0	255.0	1.98	90.3	194.9	2.16
Italy	193.7	393.8	2.03	131.5	289.6	2.20
Kazakhstan	517.0	779.9	1.51	262.9	437.5	1.66
Kyrgyzstan	806.1	1443.9	1.79	545.3	1087.4	1.99
Latvia	655.0	1156.8	1.77	353.4	718.6	2.03
Lithuania	616.0	1097.0	1.78	340.1	706.4	2.08
Luxembourg	167.1	332.7	1.99	118.6	254.9	2.15
Malta	206.0	407.7	1.98	147.8	317.0	2.15
Montenegro	510.1	922.3	1.81	415.4	829.4	2.00
Netherlands	161.3	322.0	2.00	109.1	233.5	2.14
Norway	165.8	334.3	2.02	107.1	235.1	2.20
Poland	410.4	756.0	1.84	241.9	505.6	2.09
Portugal	174.8	347.0	1.99	119.8	259.7	2.17
Republic of Moldova	750.7	1380.0	1.84	536.6	1071.5	2.00
Romania	603.5	1144.0	1.90	431.1	903.9	2.10
Russian Federation	836.1	1423.1	1.70	469.3	914.0	1.95
San Marino	242.2	516.6	2.13	155.5	322.0	2.07
Serbia	516.3	990.9	1.92	398.3	836.4	2.10
Slovakia	551.8	1048.1	1.90	360.2	758.5	2.11
Slovenia	269.2	532.9	1.98	178.0	390.6	2.19
Spain	151.0	292.4	1.94	97.3	211.5	2.17
Sweden	204.4	414.8	2.03	134.0	292.3	2.18
Switzerland	164.3	339.2	2.06	108.7	242.0	2.23
Tajikistan	710.3	1332.5	1.88	503.9	920.0	1.83
TFYR Macedonia	626.9	1228.8	1.96	490.6	1012.5	2.06
Turkey	306.7	582.7	1.90	224.8	458.2	2.04
Turkmenistan	966.4	1718.5	1.78	722.0	1335.3	1.85
The UK	176.1	334.3	1.90	110.2	227.9	2.07
Ukraine	873.3	1544.9	1.77	532.6	1065.8	2.00
Uzbekistan	858.0	1492.4	1.74	662.3	1225.1	1.85
Total mean (SD)	560.8 (231.6)	1031.5 (405.4)	1.86 (0.857)	375.3 (164.6)	751.3 (313.6)	2.03 (0.094)

Mean CVD rates calculated using both ESPs were significantly lower in females than males (mean rate males, ASMR76 = 560.8, ASMR13 = 1031.5; mean rate females, ASMR76 = 375.3, ASMR13 = 751.3, *P* < 0.001), but females had a significantly greater proportional difference in rates when comparing the ASMR76s and ASMR13s (mean rate difference; males = 1.86, females = 2.03, *P* < 0.001). Spearman’s rank coefficient showed that ranks of countries by ASMRs calculated using the two ESPs were different for both sexes (Spearman’s rho for men 0.995, *P* < 0.001; Spearman’s rho for women 0.97, *P* < 0.001).

The largest changes in ranking were found for males in Kazakhstan which was six places lower when the ranking was compared between ASMR13 and ASMR76. In women, Ukraine was four places lower. In both sexes, Central Asian and Eastern European countries moved the most number of ranked places. In men, Central Asian and Eastern European countries generally moved down the ranking, and for women, these countries generally moved up. For both sexes, the top two and bottom three countries remained the same for rates calculated using both ESP (Table 3).

**Table 3:** Ranking number for countries from lowest to highest cardiovascular disease mortality rates (per 100,000), by latest available year

Country	Males		Change in ranking	Females		Change in ranking
	1976 ESP	2013 ESP		1976 ESP	2013 ESP	
Albania	36	36	0	36	34	2
Armenia	35	34	1	34	36	− 2
Austria	18	18	0	18	20	− 2
Azerbaijan	40	38	2	38	44	− 6
Belarus	49	48	1	48	35	13
Belgium	11	11	0	11	9	2
Bosnia and Herzegovina	28	30	− 2	30	38	− 8
Bulgaria	43	43	0	43	45	− 2
Croatia	26	27	− 1	27	29	− 2
Cyprus	16	16	0	16	19	− 3
Czech Republic	25	25	0	25	27	− 2
Denmark	8	8	0	8	5	3
Estonia	31	31	0	31	28	3
Finland	21	20	1	20	15	5
France	2	2	0	2	1	1
Georgia	30	29	1	29	30	− 1
Germany	19	19	0	19	21	− 2
Greece	22	21	1	21	23	− 2
Hungary	29	32	− 3	32	31	1
Iceland	17	17	0	17	16	1
Ireland	15	15	0	15	13	2
Israel	1	1	0	1	2	− 1
Italy	12	12	0	12	12	0
Kazakhstan	34	28	6	28	24	4
Kyrgyzstan	46	47	− 1	47	49	− 2
Latvia	42	41	1	41	33	8
Lithuania	39	39	0	39	32	7
Luxembourg	7	5	2	5	10	− 5
Malta	14	13	1	13	17	− 4
Montenegro	32	33	− 1	33	39	− 6
Netherlands	4	4	0	4	6	− 2
Norway	6	6	0	6	7	− 1
Poland	27	26	1	26	26	0
Portugal	9	10	− 1	10	11	− 1
Republic of Moldova	45	45	0	45	48	− 3
Romania	38	40	− 2	40	41	− 1
Russia Federation	47	46	1	46	42	4
San Marino	20	22	− 2	22	18	4
Serbia	33	35	− 2	35	40	− 5
Slovakia	37	37	0	37	37	0
Slovenia	23	23	0	23	22	1
Spain	3	3	0	3	3	0
Sweden	13	14	− 1	14	14	0
Switzerland	5	9	− 4	9	8	1
Tajikistan	44	44	0	44	43	1
TFYR Macedonia	41	42	− 1	42	46	− 4
Turkey	24	24	0	24	25	− 1
Turkmenistan	51	51	0	51	51	0
The UK	10	7	3	7	4	3
Ukraine	50	50	0	50	47	3
Uzbekistan	48	49	− 1	49	50	− 1

*1 = lowest, 51 = highest

A majority of countries in both sexes showed a negative ASMR trend calculated using both ESPs. Joinpoint analyses showed no difference in the direction of trend between ASMRs. There were four countries (Albania, Slovenia, Tajikistan, and Turkey) in men and eight countries (Azerbaijan, Belarus, Montenegro, San Marino, Serbia, Slovakia, Slovenia, and Turkey) in women that have a continuous linear trend (no joinpoints identified).

In men, seven countries (San Marino, Austria, Belarus, Azerbaijan, Lithuania, Romania, and Hungary) had a greater decrease in the trend for ASMR13 than ASMR76. This was found in three countries for women (Belarus, Latvia, and Russia). A greater increase in trend in ASMR13 than ASMR76 was found in six countries for women (Albania, Bulgaria, Azerbaijan, Turkey, Kyrgyzstan, and Turkmenistan) and in one country for men (TFYR Macedonia). Four countries (Bulgaria, Russian Federation, Ukraine, and Uzbekistan) had a greater increase in trend for ASMR76 than ASMR13 in men and two in women (Kazakhstan and Uzbekistan) (Table 4).

**Table 4:** Average annual percentage changes and joinpoint analysis by country and sex by recent available year in males

	Average annual percentage change (AAPC)		Joinpoint annual percentage change (APC) and end year for each segment in the best fitting model									
Males			Segment 1		Segment 2		Segment 3		Segment 4		Segment 5	
Country												
Albania	ESP 76	− 0.3 (− 1.3, 0.7)										
	ESP 13	− 0.1 (− 1.2, 0.9)										
Armenia	ESP 76	0 (− 0.7, 0.7)	1981	2.7^^^	1993	− 5.5^	2000	17.6^^^	2003	− 3.9^		
	ESP 13	0 (− 0.9, 0.8)	1981	2.5^^^	1993	− 6.4^	2000	22.6^^^	2003	− 4.4^^^		
Austria	ESP 76	− 2.8^^^ (− 3.4, − 2.3)	1965	18.1^^^	1971	− 3.6^^^						
	ESP 13	− 2.9 (− 3.5, − 2.3)	1965	21.2^^^	1971	− 3.7^^^						
Azerbaijan	ESP 76	− 0.4^^^ (− 0.8, − 0.1)	1981	0.7	1995	− 1.7^^^						
	ESP 13	− 0.5^^^ (− 0.8, − 0.1)	1981	− 0.2	2003	− 4.6^^^						
Belarus	ESP 76	− 0.5 (− 1, 0.1)	1981	3.9	1985	− 3.8^^^	1992	7.1	1996	− 2.1^^^		
	ESP 13	− 0.7 (− 1.2, − 0.2)	1981	4.7	1985	− 4.8^^^	1992	7.2	1996	− 2.2^^^		
Belgium	ESP 76	− 2.6^ (− 2.9, − 2.3)	1965	4.4	1971	− 2.9^						
	ESP 13	− 2.3 (− 2.6, − 2.1)	1965	4.5	1971	− 2.7^						
Bosnia and Herzegovina	ESP 76	− 0.7 (− 1.7, 0.3)	1985	8.5^^^	1988	− 1.1^^^						
	ESP 13	− 0.4 (− 1.4, 0.7)	1985	9.3	1988	− 0.9^						
Bulgaria	ESP 76	0.8^^^ (0.5, 1.2)	1965	24.1^^^	1969	1.5^^^	1998	− 1.8^^^				
	ESP 13	0.7^^^ (0.4, 1.1)	1965	23.7^^^	1969	1.4^^^	1998	− 1.8^^^				
Croatia	ESP 76	− 1.9^^^ (− 2.3, − 1.4)	1985	− 1.8^^^	1995	5.8	1998	− 4.0^^^				
	ESP 13	− 1.7^^^ (− 2.1, − 1.3)	1985	− 1.9^^^	1995	5.8	1998	− 3.7^^^				
Cyprus	ESP 76	− 3.8^^^ (− 4.7, − 2.8)										
	ESP 13	− 3.3^^^ (− 4.6, − 2.1)										
Czech Republic	ESP 76	− 3.0^^^ (− 3.2, − 2.9)	1986	− 0.5	1990	− 3.4^	2000	− 1.1	2003	− 5.1^^^	2013	− 2.5^^^
	ESP 13	− 2.8^^^ (− 2.9, − 2.6)	1986	− 0.5	1990	− 3.1^^^	2000	− 0.2	2003	− 5.3^^^	2007	− 2.4^^^
Denmark	ESP 76	− 2.2^^^ (− 2.6, − 1.9)	1965	2.3^^^	1973	− 1.5^^^	1991	− 3.7^^^	2003	− 6.0^^^		
	ESP 13	− 2.1^^^ (− 2.4, − 1.8)	1965	2.9^^^	1972	− 1.4^^^	1991	− 3.4^^^	2003	− 5.9^^^		
Estonia	ESP 76	− 2.0^^^ (− 2.3, − 1.7)	1981	− 0.9^^^	1991	3.2	1994	− 5.5	1997	− 2.2^^^	2012	− 5.2^^^
	ESP 13	− 2.0^^^ (− 2.3, − 1.8)	1981	− 0.8^^^	1994	− 2.4^^^	2007	− 4.9^^^				
Finland	ESP 76	− 2.6^^^ (− 2.8, − 2.4)	1965	5.7^^^	1970	− 2.2^^^	1987	− 3.3^^^				
	ESP 13	− 2.3^^^ (− 2.5, − 2.1)	1965	5.9^^^	1970	− 2.1^^^	1990	− 3.1^^^				
France	ESP 76	− 2.4^^^ (− 2.7, − 2.1)	1965	13.7^^^	1969	− 2.0^^^	1986	− 6.8	1989	− 1.9^^^	2011	− 4.4^^^
	ESP 13	− 2.3^^^ (− 2.6, − 2)	1965	14.2^^^	1969	− 1.9^^^	1986	− 6.7	1989	− 1.7^^^	2002	− 4.3^^^
Georgia	ESP 76	− 1.7^^^ (− 2.5, − 0.9)	1981	− 0.2	2007	− 17.9	2011	9.5				
	ESP 13	− 1.8^^^ (− 2.6, − 0.9)	1981	− 0.1	2005	− 8.7^						
Germany	ESP 76	− 3.6^^^ (− 3.8, − 3.4)	1990	− 3.2^^^	2003	− 4.6^^^	2011	1.4				
	ESP 13	− 3.4^^^ (− 3.6, − 3.3)	1990	− 3.0^^^	2003	− 4.6^^^	2011	2				
Greece	ESP 76	− 0.4^^^ (− 0.8, − 0.1)	1965	23.2^^^	1968	1.3^^^	1987	− 1.0^^^	2002	− 4.1^^^		
	ESP 13	− 0.3 (− 0.7, 0.1)	1965	23.2^^^	1968	1.5^^^	1987	− 0.8^^^	2002	− 4.5^^^		
Hungary	ESP 76	− 0.6^^^ (− 0.9, − 0.3)	1965	10.9^^^	1970	0.2	1992	−^^^2.3^				
	ESP 13	− 0.7^^^ (-1, -0.4)	1965	10.7^^^	1970	−0.2	1993	− 2.2^				
Iceland	ESP 76	− 2.6^^^ (− 2.8, − 2.4)	1965	5.7^^^	1970	− 2.2^^^	1987	− 3.3^^^				
	ESP 13	− 2.3^^^ (− 2.5, − 2.1)	1965	5.9^^^	1970	− 2.1^^^	1990	− 3.1^^^				
Ireland	ESP 76	− 2.3^^^ (− 2.7, − 1.9)	1965	7.7^^^	1969	− 0.4	1982	− 2.8^^^	1998	− 5.4^^^		
	ESP 13	− 2.2^^^ (− 2.5, − 1.8)	1965	7.8^^^	1969	− 0.4	1982	− 2.7^^^	1998	− 5.1^^^		
Israel	ESP 76	− 3.9^^^ (− 4.2, − 3.6)	1975	− 2.8^^^	1990	0.3	1994	− 10.2^^^	1997	− 4.7^^^		
	ESP 13	− 3.7^^^ (− 3.9, − 3.4)	1975	− 2.6^^^	1990	1	1994	− 10.2^^^	1997	− 4.5^^^		
Italy	ESP 76	− 2.4^^^ (− 2.7, − 2.1)	1965	11.0^^^	1969	− 1.4^^^	1983	− 3.2^^^				
	ESP 13	− 2.2^^^ (− 2.5, − 1.9)	1965	11.3^^^	1969	− 1.3^^^	1983	− 3.0^^^				
Kazakhstan	ESP 76	0.2 (− 0.6, 1.1)	1981	0.4	1991	7.7	1994	0.3	2007	− 11.3^^^	2012	− 25.3^^^
	ESP 13	0.1 (− 0.8, 1)	1981	0.7	1991	6.3	1994	0.5	2007	− 11.9^^^	2010	− 29.7^^^
Kyrgyzstan	ESP 76	0.9^^^ (0.7, 1.2)	1981	0.1	1991	5.7	1994	0.4^^^				
	ESP 13	0.9^^^ (0.7, 1.2)										
Latvia	ESP 76	− 0.9^^^ (− 1.2, − 0.5)	1980	− 0.8^^^	1991	9.3^^^	1994	− 9.2^^^	1997	− 0.3	2012	− 3.9^^^
	ESP 13	− 1.0^^^ (− 1.3, − 0.8)	1980	− 1.0^^^	1991	6.8	1994	− 7.6	1997	− 0.3	2005	− 3.3^^^
Lithuania	ESP 76	− 0.3^^^ (− 0.6, − 0.1)	1981	1.1^^^	1994	− 2.4^^^	2000	1.5	2006	− 3.0^^^		
	ESP 13	− 0.4^^^ (− 0.6, − 0.2)	1981	0.6	1994	− 0.9^^^						
Luxembourg	ESP 76	− 2.5^^^ (− 2.9, − 2.1)	1965	8.8^^^	1971	− 1.5^^^	1985	− 3.9^^^				
	ESP 13	− 2.2^^^ (− 2.6, − 1.8)	1965	9.7^^^	1971	− 1.2^^^	1985	− 3.7^^^				
Malta	ESP 76	−2.9^^^ (− 3.5, − 2.3)	1965	5.8^^^	1981	− 20.4	1984	− 3.5^^^				
	ESP 13	− 2.7^^^ (− 3.4, − 2.1)	1965	6.7^^^	1981	− 22.4	1984	− 3.2^^^				
Montenegro	ESP 76	− 0.7 (− 2.1, 0.7)	2000	−4.7	2002	3	2006	−5.8^^^				
	ESP 13	− 0.5 (− 2, 0.9)	2000	− 4.8	2002	3.5	2006	− 6.0^^^				
Netherlands	ESP 76	− 2.2^^^ (− 2.5, − 1.9)	1965	− 1.9	1967	12.6^^^	1970	− 1.3^^^	1985	− 2.4^^^	2013	− 5.1^^^
	ESP 13	− 2.0^^^ (− 2.3, − 1.7)	1965	− 2.4	1967	13.1^^^	1970	− 1.3^^^	1985	− 2.1^^^	2000	− 4.8^^^
Norway	ESP 76	− 2.1^^^ (− 2.5, −1.8)	1965	9.9^^^	1970	− 1.0^^^	1988	− 2.9^^^	1998	− 5.1^^^		
	ESP 13	− 1.9^^^ (− 2.2, − 1.5)	1965	11.3^^^	1970	− 1.2^^^	1995	− 4.6^^^				
Poland	ESP 76	− 0.7^^^ (− 1.2, − 0.3)	1965	5.6^^^	1971	1.4^^^	1991	− 3.6^^^	2002	− 2.4^^^		
	ESP 13	− 0.7^^^ (− 1.1, − 0.3)	1965	5.4^^^	1971	1.5^^^	1990	− 2.9^^^				
Portugal	ESP 76	− 2.2^^^ (− 2.8, − 1.6)	1965	0.2	1969	33.3^^^	1972	− 2.0^^^	1993	− 4.5^^^		
	ESP 13	− 2.0^^^ (− 2.6, − 1.4)	1965	0.1	1969	35.0^^^	1972	− 1.8^^^	1993	− 4.4^^^		
Republic of Moldova	ESP 76	0.1 (− 0.4, 0.7)	1981	− 0.3	1985	− 4.9^^^	1992	10.5^^^	1996	0.6	2013	− 2.8^^^
	ESP 13	0.1 (− 0.5, 0.7)	1981	0.5	1985	− 6.3^^^	1992	11.7^^^	1996	1	2003	− 3.2^^^
Romania	ESP 76	− 0.2 (− 0.5, 0.1)	1969	0.8^^^	1997	− 2.4^^^						
	ESP 13	− 0.3^^^ (− 0.5, 0)	1969	0.7^^^	1997	− 2.3^^^						
Russian Federation	ESP 76	0.3 (0, 0.7)	1980	− 1.1^^^	1991	10.2^^^	1994	− 6.7	1997	4.0^^^	2011	− 3.8^^^
	ESP 13	0 (−0.3, 0.4)	1980	− 1.1^^^	1991	7.4	1994	− 5.7	1997	2.8^^^	2004	− 4.0^^^
San Marino	ESP 76	− 3.7 (−12.7, 6.2)										
	ESP 13	− 4.6 (− 13.9, 5.7)										
Serbia	ESP 76	− 2.5^^^ (− 2.8, − 2.2)										
	ESP 13	− 2.4^^^ (− 2.7, − 2.1)										
Slovakia	ESP 76	− 1.2^^^ (− 1.5, − 0.8)	1992	2.6	1995	− 1.2^^^	2006	− 3.2^^^				
	ESP 13	− 0.8^^^ (− 1.2, − 0.4)	1992	3.6	1995	− 0.9^^^	2006	− 3.2^^^				
Slovenia	ESP 76	− 3.4^^^ (− 3.6, − 3.2)										
	ESP 13	− 3.2^^^ (− 3.4, − 3)										
Spain	ESP 76	− 2.6^^^ (− 3, − 2.3)	1965	− 30.8^^^	1968	16.0^^^	1973	− 3.0^^^				
	ESP 13	− 2.6^^^ (− 2.9, − 2.2)	1965	− 34.8^^^	1968	16.8^^^	1973	− 2.9^^^				
Sweden	ESP 76	− 2.1^^^ (− 2.4, − 1.8)	1965	4.5^^^	1971	− 0.3	1982	− 2.8^^^	1998	− 3.7^^^		
	ESP 13	− 1.9^^^ (− 2.2, − 1.6)	1965	4.6^^^	1971	− 0.4	1982	− 2.5^^^	1998	− 3.4^^^		
Switzerland	ESP 76	− 2.5^^^ (− 2.7, − 2.2)	1965	− 3.5	1967	7.8^^^	1970	− 0.8^^^	1980	− 2.8^^^	2013	− 3.7^^^
	ESP 13	− 2.3^^^ (− 2.5, − 2.1)	1965	− 3.9	1967	8.7^^^	1970	− 0.8^^^	1980	− 2.7^^^	1997	− 3.5^^^
Tajikistan	ESP 76	1.5^^^ (1, 2)										
	ESP 13	1.8^^^ (1.3, 2.3)										
TFYR Macedonia	ESP 76	0 (− 0.3, 0.4)	1991	0.9^^^	2003	− 1.7^^^						
	ESP 13	0.4^^^ (0, 0.8)	1991	1.4^^^	2003	− 1.5^^^						
Turkey	ESP 76	1.1 (− 1.5, 3.8)										
	ESP 13	1.1 (− 1.5, 3.8)										
Turkmenistan	ESP 76	1.4 (− 0.2, 3)	1981	0.5	1989	12.5	1992	− 5.3^^^				
	ESP 13	1.4 (0, 3)	1981	0.7	1989	11.5	1992	− 4.7^^^				
The UK	ESP 76	− 2.7^^^ (− 3, − 2.4)	1965	8.6^^^	1969	− 1.1^^^	1979	− 2.9^^^	1999	− 5.2^^^		
	ESP 13	− 2.6^^^ (− 2.9, − 2.3)	1965	9.1^^^	1969	− 1.2^^^	1979	− 2.7^^^	2001	− 5.5^^^		
Ukraine	ESP 76	0.7^^^ (0.3, 1.1)	1981	− 1.8^^^	1991	7.1^^^	1995	− 2.2	1998	2.3^^^	2012	− 3.1^^^
	ESP 13	0.5^^^ (0.2, 0.9)	1981	0.7	1985	− 3.8^^^	1991	7.5	1994	1.1^^^	2006	− 3.1^^^
Uzbekistan	ESP 76	0.7^^^ (0.3, 1.1)	1981	− 1.8^^^	1991	7.1^^^	1995	− 2.2	1998	2.3^^^	2012	− 3.1^^^
	ESP 13	0.5^^^ (0.2, 0.9)	1981	0.7	1985	− 3.8^^^	1991	7.5	1994	1.1^^^	2006	− 3.1^^^

*AAPC* the average annual percentage change over the entire period of available data, *APC* annual percentage change over each identified joinpoint segment

^^^Values are significantly different to zero

Differences in the number of joinpoint segments between rates from both ESPs were found for five countries in men (Georgia, Lithuania, Norway, and Poland) and four countries (Albania, Estonia, Kyrgyzstan, and Lithuania) in women. A number of countries showed a plateau in recent trends, as defined by the most recent segment showing either a less steep decline than the preceding segment, no significant difference to zero, or an increase. These plateaus were identified at all times in trends of both ASMRs except for men in Georgia amongst whom a plateau found in the trend of ASMR76 was not found when using ASMR13 (Table 5).

**Table 5:** Average annual percentage changes and joinpoint analysis by country and sex by recent available year in females

Country	Average annual percentage change (AAPC)		Joinpoint annual percentage change (APC) and end year for each segment in the best fitting model									
Females			Segment 1		Segment 2		Segment 3		Segment 4		Segment 5	
Country												
Albania	ESP 76	0.1 (− 1.2, 1.4)	1987	− 4.9	1993	2.2^^^						
	ESP 13	0.2 (−1.1, 1.6)										
Armenia	ESP 76	− 0.3 (− 1, 0.3)	1981	2.2^^^	1993	− 4.4^^^	2000	12.9^^^	2003	− 4.3^^^		
	ESP 13	− 0.1 (− 0.8, 0.5)	1981	2.2^^^	1993	− 4.6^^^	2000	15.2^^^	2003	− 4.3^^^		
Austria	ESP 76	− 1.9^^^ (− 2.4, − 1.4)	1965	1.4	1983	− 3.2^^^						
	ESP 13	− 1.4^^^ (− 1.9, − 0.9)	1965	2.0^^^	1984	− 3.0^^^						
Azerbaijan	ESP 76	0.4^^^ (0.1, 0.7)										
	ESP 13	0.5^^^ (0.1, 0.9)										
Belarus	ESP 76	− 0.5 (− 1, 0.1)	1981	3.9	1985	− 3.8	1992	7.1	1996	− 2.1^^^		
	ESP 13	− 0.7^^^ (− 1.2, − 0.2)	1981	4.7	1985	− 4.8^^^	1992	7.2	1996	− 2.2^^^		
Belgium	ESP 76	− 2.5^^^ (− 2.7, − 2.3)	1965	7.8^^^	1969	− 2.6^^^	2004	− 4.3^^^				
	ESP 13	− 2.3^^^ (− 2.5, − 2.1)	1965	7.7^^^	1969	− 2.4^^^	2004	− 4.3^^^				
Bosnia and Herzegovina	ESP 76	− 0.8 (− 1.7, 0.1)	1985	4.6	1989	− 1.3^^^						
	ESP 13	− 0.4 (− 1.3, 0.5)	1985	5	1989	− 0.9^^^						
Bulgaria	ESP 76	0.1 (− 0.2, 0.4)	1965	22.6^^^	1969	0.5^^^	1998	− 2.1^^^				
	ESP 13	0.2 (− 0.1, 0.5)	1965	22.6^^^	1969	0.6^^^	1998	− 2.0^^^				
Croatia	ESP 76	− 2.0^^^ (− 2.4, − 1.6)	1985	− 2.0^^^	1995	5.7	1998	− 4.0^^^				
	ESP 13	− 1.7^^^ (− 2.1, − 1.3)	1985	− 1.9^^^	1995	6	1998	− 3.6^^^				
Cyprus	ESP 76	− 2.9^^^ (− 4.8, − 0.8)										
	ESP 13	− 2.2^^^ (− 4.4, 0)										
Czech Republic	ESP 76	− 2.8^^^ (− 2.9, − 2.6)	1986	− 2.3^^^	2003	− 3.8^^^						
	ESP 13	− 2.5^^^ (− 2.7, − 2.3)	1986	− 2.0^^^	2003	− 3.6^						
Denmark	ESP 76	− 2.3^^^ (− 2.6, − 2.1)	1965	3.2^^^	1970	− 1.7^^^	1993	− 3.4^^^	2003	− 5.6^^^		
	ESP 13	− 2.2^^^ (− 2.5, − 2)	1965	3.2^^^	1970	− 1.6^^^	1993	− 3.3^^^	2003	− 5.4^^^		
Estonia	ESP 76	− 2.7^^^ (− 3, − 2.4)	1981	1.1	1985	− 2.6^^^	1991	1.1	1994	− 3.8^^^		
	ESP 13	− 2.5_^_ (− 2.8, − 2.3)	1981	1.2	1985	− 2.3^^^	2002	− 3.9^^^				
Finland	ESP 76	− 2.9^^^ (− 3, − 2.7)	1965	− 3.1	1967	11.6^^^	1970	− 4.7^^^	1977	− 2.2^^^	2013	− 3.5^^^
	ESP 13	− 2.9^^^ (− 3, − 2.7)	1965	− 3.3	1967	11.5^^^	1970	− 4.6^^^	1977	− 2.0^^^	2013	− 3.3^^^
France	ESP 76	− 2.6^^^ (− 2.8, − 2.3)	1965	14.9^^^	1969	− 2.3^^^	1986	− 5.8	1990	− 2.0^^^	2011	− 4.7^^^
	ESP 13	− 2.4^^^ (− 2.7, − 2.1)	1965	15.0^^^	1969	− 2.0^^^	1986	− 5.8	1990	− 1.9^^^	2011	− 4.8^^^
Georgia	ESP 76	− 2.2^^^ (− 3, − 1.5)	1981	− 0.5	2004	− 7.9^^^						
	ESP 13	− 2.2^^^ (− 3, − 1.4)	1981	− 0.6	2004	− 7.8^^^						
Germany	ESP 76	− 3.0^^^ (− 3.2, − 2.9)	1990	− 3.0^^^	2000	− 0.7	2003	− 5.3^^^	2006	− 3.7^^^	2013	− 0.4
	ESP 13	− 2.8^^^ (− 3, − 2.6)	1990	− 2.8^^^	2000	− 0.1	2003	− 5.3^^^	2006	− 3.5^^^	2013	− 0.3
Greece	ESP 76	− 0.5^^^ (− 0.9, − 0.2)	1965	26.7^^^	1968	1.0^^^	1987	− 0.9^^^	2004	− 5.4^^^		
	ESP 13	− 0.2 (− 0.6, 0.1)	1965	26.1^^^	1968	1.4^^^	1986	− 0.5^^^	2004	− 5.5^^^		
Hungary	ESP 76	− 1.2^^^ (− 1.4, − 0.9)	1965	9.3^^^	1970	− 0.7^^^	1993	− 2.5^^^				
	ESP 13	− 1.1^^^ (− 1.3, − 0.9)	1965	9.1^^^	1970	− 0.8^^^	1996	− 2.4^^^				
Iceland	ESP 76	− 2.7^^^ (− 3, − 2.4)	1965	10.4^^^	1969	− 1.1	1978	− 3.0^^^	1998	− 5.0^^^		
	ESP 13	− 2.5^^^ (− 2.8, − 2.2)	1965	14.0^^^	1968	0	1976	− 2.7^^^	1998	− 4.7^^^		
Ireland	ESP 76	− 2.7^^^ (− 3, − 2.4)	1965	10.4^^^	1969	− 1.1	1978	− 3.0^^^	1998	− 5.0^^^		
	ESP 13	− 2.5^^^ (− 2.8, − 2.2)	1965	14.0^^^	1968	0	1976	− 2.7^^^	1998	− 4.7^^^		
Israel	ESP 76	− 4.5^^^ (− 4.7, − 4.2)	1975	− 3.2^^^	1999	− 13.6	2013	− 5.2				
	ESP 13	− 4.2^^^ (− 4.4, − 3.9)	1975	− 2.9^^^	1995	− 8.0^^^	1999	− 4.4^^^				
Italy	ESP 76	− 2.7^^^ (− 2.9, − 2.5)	1965	12.7^^^	1968	− 0.8	1976	− 3.1^^^				
	ESP 13	− 2.4^^^ (− 2.7, − 2.2)	1965	12.4^^^	1968	− 0.4	1976	− 2.9^^^				
Kazakhstan	ESP 76	0.2 (− 0.6, 1.1)	1981	0.4	1991	7.7	1994	0.3	2007	− 11.3^^^	2012	− 25.3^^^
	ESP 13	0.1 (− 0.8, 1)	1981	0.7	1991	6.3	1994	0.5	2007	− 11.9^^^	2012	− 29.7^^^
Kyrgyzstan	ESP 76	1.0^^^ (0.7, 1.2)	1981	0.1	1992	6.5	1995	− 4	1998	4.8	2013	− 0.5
	ESP 13	1.2^^^ (0.9, 1.4)										
Latvia	ESP 76	− 1.6^^^ (− 1.9, − 1.4)	1980	− 1.2^^^	2003	− 3.7^^^						
	ESP 13	− 1.7^^^ (− 1.9, − 1.5)	1980	− 1.3^^^	2003	− 3.5^^^						
Lithuania	ESP 76	− 1.2^^^ (− 1.4, − 1)	1981	− 0.2	1995	− 1.8^^^						
	ESP 13	− 1.1 (− 1.3, − 0.9)	1981	1.6	1985	− 0.9^^^	2006	− 3.2^^^				
Luxembourg	ESP 76	− 2.7^^^ (− 3, − 2.3)	1965	7.4^^^	1971	− 1.2^^^	1984	− 4.4^^^	2000	3.2	2013	− 5.6^^^
	ESP 13	− 2.4^^^ (− 2.8, − 2.1)	1965	18.4^^^	1968	0	1983	− 4.3^^^	2000	3.4	2013	− 5.3^^^
Malta	ESP 76	− 3.4^^^ (− 3.9, − 2.8)	1965	5.3^^^	1981	− 21.9^^^	1984	− 3.7^^^				
	ESP 13	− 3.2^^^ (− 3.8, − 2.6)	1965	6.0^^^	1981	− 23.6^^^	1984	− 3.4^^^				
Montenegro	ESP 76	− 0.5 (− 1.9, 0.9)										
	ESP 13	− 0.2 (− 1.6, 1.2)										
Netherlands	ESP 76	− 2.3^^^ (− 2.5, − 2)	1965	− 5.6	1967	16.6^^^	1970	− 3.1^^^	1980	− 1.9^^^	2013	− 4.1^^^
	ESP 13	− 2.2^^^ (− 2.4, − 1.9)	1965	− 5.8	1967	16.6^^^	1970	− 3.1^^^	1980	− 1.9^^^	2013	− 3.9^^^
Norway	ESP 76	− 2.2^^^ (− 2.5, − 1.9)	1965	11.5^^^	1970	− 2.0^^^	1999	− 4.5^^^				
	ESP 13	− 2.0^^^ (− 2.3, − 1.7)	1965	− 1	1967	16.7^^^	1970	− 1.9^^^	1999	− 4.2^^^		
Poland	ESP 76	− 1.1^^^ (− 1.4, − 0.7)	1965	4.9^^^	1970	0.9^^^	1991	− 3.2^^^				
	ESP 13	− 0.9^^^ (− 1.2, − 0.5)	1965	5.2^^^	1970	1.0^^^	1991	− 2.9^^^				
Portugal	ESP 76	− 2.3^^^ (− 2.9, − 1.7)	1965	0.1	1969	33.6^^^	1972	− 2.2^^^	1996	− 4.9^^^		
	ESP 13	− 2.0^^^ (− 2.6, − 1.4)	1965	0.5	1969	34.1^^^	1972	− 1.7^^^	1995	− 4.6^^^		
Republic of Moldova	ESP 76	− 0.1 (− 0.7, 0.5)	1981	1	1985	− 6.4^^^	1992	9.9^^^	1996	1	2013	− 3.2^^^
	ESP 13	− 0.1 (− 0.7, 0.5)	1981	1.6	1985	− 7.6^^^	1992	10.8^^^	1996	1.6	2013	− 3.1^^^
Romania	ESP 76	− 0.9^^^ (− 1.1, − 0.6)	1969	0.7^^^	1985	− 1.0^^^	2003	− 3.7^^^				
	ESP 13	− 0.8^^^ (− 1, − 0.5)	1969	1.1^^^	1984	− 1.0^^^	2003	− 3.3^^^				
Russian Federation	ESP 76	− 0.2 (− 0.5, 0.1)	1980	− 1.0^^^	1991	5.8	1994	− 4.1	1997	2.4^^^	2011	− 4.0^^^
	ESP 13	− 0.3^^^ (− 0.6, 0)	1980	− 0.5	1991	0.6^^^	2005	− 4.6^^^				
San Marino	ESP 76	0.9 (− 19.6,26.7)										
	ESP 13	0.9 (− 19.6, 26.7)										
Serbia	ESP 76	− 2.7^^^ (− 3, − 2.5)										
	ESP 13	− 2.5^^^ (− 2.7, − 2.2)										
Slovakia	ESP 76	− 2.7^^^ (− 3, − 2.5)										
	ESP 13	− 2.5^^^ (− 2.7, − 2.2)										
Slovenia	ESP 76	− 3.4^^^ (− 3.6, − 3.2)										
	ESP 13	− 3.2^^^ (− 3.4, − 2.9)										
Spain	ESP 76	− 3.0^^^ (− 3.3, − 2.7)	1965	24.2^^^	1969	− 2.7^^^	1990	− 3.9^^^				
	ESP 13	− 2.7^^^ (− 3.1, − 2.4)	1965	24.7^^^	1969	− 2.3^^^	1990	− 3.8^^^				
Sweden	ESP 76	− 2.2^^^ (− 2.4, − 2)	1965	5.3^^^	1970	− 1.7^^^	1985	− 2.8^^^				
	ESP 13	− 2.1^^^ (− 2.2, − 1.9)	1965	5.3^^^	1970	− 1.6^^^	1985	− 2.6^^^				
Switzerland	ESP 76	− 2.7^^^ (− 2.9, − 2.5)	1965	− 4.1	1967	10.6^^^	1970	− 3.0^^^				
	ESP 13	− 2.5^^^ (− 2.7, − 2.3)	1965	− 3.7	1967	11.0^^^	1970	− 2.8^^^				
Tajikistan	ESP 76	− 2.7^^^ (− 2.9, − 2.5)	1965	− 4.1	1967	10.6^^^	1970	− 3.0^^^				
	ESP 13	− 2.5^^^ (− 2.7, − 2.3)	1965	− 3.7	1967	11.0^^^	1970	− 2.8^^^				
TFYR Macedonia	ESP 76	− 2.7^^^ (− 2.9, − 2.5)	1965	− 4.1	1967	10.6^^^	1970	− 3.0^^^				
	ESP 13	− 2.5^^^ (− 2.7, − 2.3)	1965	− 3.7	1967	11.0^^^	1970	− 2.8^^^				
Turkey	ESP 76	0.1 (− 4.8, 5.2)										
	ESP 13	0.5 (− 4.6, 6)										
Turkmenistan	ESP 76	1.7 (− 0.2, 3.7)	1981	0.5	1989	15.3	1992	− 6.1^^^				
	ESP 13	1.8 (− 0.3, 3.8)	1981	0.5	1989	15.9	1992	− 6.4^^^				
The UK	ESP 76	– 2.7^^^ (− 3, − 2.4)	1965	13.2^^^	1969	− 2.6^^^	2001	− 5.5^^^				
	ESP 13	– 2.5^^^ (− 2.8, − 2.3)	1965	13.1^^^	1969	− 2.5^^^	2002	− 5.4^^^				
Ukraine	ESP 76	0.1 (− 0.2, 0.4)	1981	1.6	1985	− 4.3^^^	1991	8.1	1994	0.4	2012	− 2.7^^^
	ESP 13	0.1 (− 0.2, 0.4)	1981	− 0.4	1988	− 8.1	1991	9.1^^^	1994	0.5	2012	− 2.4^^^
Uzbekistan	ESP 76	1.5^^^	(0.9, 2)	1981	0.5	1990	7.4^^^	1994	− 0.7^^^			
	ESP 13	1.4^^^	(0.9, 2)	1981	0.6	1990	7.5^^^	1994	− 0.9^^^			

*AAPC* refers to the average annual percentage change over the entire period of available data, *APC* refers to annual percentage change over each identified joinpoint segment

^^^Values are significantly different to zero

## Discussion

The 2013 ESP changes the relative burden of CVD mortality rates for European countries by sex. The 1976 ESP rates are half as high as those calculated using 2013 ESP in countries for both sexes, and the ranking of countries by CVD ASMR changed when calculating ASMRs using the different standard populations. Despite largely similar trends between ASMR13 and ASMR76 for all countries by sex, there were some differences in trends in ASMRs calculated using different ESPs.

Joinpoint analyses allowed us to compare trends in the log of ASMRs calculated using the old and new ESPs. This demonstrated that for most countries the direction and intensity of the trend in the two ASMRs were similar, although some differences were apparent. In particular, a number of countries showed a difference in the intensity of the trend over the entire period, when comparing ESP76 to ESP13, although no country was found to have a change in the direction of the trend for either sex. Differences in intensity were more pronounced in countries when comparing the most recent trend, with one country demonstrating a change in the direction of the most recent segment.

Limitations of this research are that there is a variability of global coverage and data quality [11]. Developed countries use the vital record system whereas developing countries use verbal autopsy, which are generally weaker and not standardised [12, 13]. Furthermore, the results in this study may not be generalizable to countries outside of the WHO Europe Member States. However, findings are representable and internally valid because data were comparable between ESPs since the same data were used.

To our knowledge, this is the first paper to investigate trends in CVD mortality for all European countries. It is also the first to compare ASMRs calculated using ESP13 and ESP76 [2]. Our finding that ASMR76 were half as large as ASMR13s was similar to previous work investigating deaths from coronary heart disease (CHD) rather than CVD as done here [2]. We found no paper that had investigated the difference between trends in ASMRs calculated using ESP13 and ESP76 in total CVD or subtype, although trends in CVD agreed with previous analysis on trends in CHD ASMRs [11] in most countries. Differences in results between studies show a decrease in CHD ASMR in Bulgaria found in the previous [11] compared to an increase in CVD ASMR in the present study. These differences may be due to garbage codes, defined as incomplete registration of death and sex/age in the mortality data for CVD [14]. The WHO reported that Bulgaria had a large amount of garbage codes included for subgroups in CVD [15].

Such changes in standard populations can be confusing for policy makers and public alike, unless clarity is provided in the effect of such changes. We have demonstrated in this paper that the relative description of the burden of CVD mortality between countries occurs when changing the standard population used to calculate age-standardised rates. In addition, in some countries, the intensity and, in one country, the direction, of the most recent trend in CVD ASMRs was altered by the introduction of a new standard population, despite no change in the mortality and population data used to calculate them. Such changes can be misleading and care must be taken not to compare between analyses using different standard populations. ASMRs produce an estimate of mortality that relates to a standard population; this means it is only a useful measure when used comparatively between estimates using the same population. Rates calculated using the actual population are better when calculating an absolute mortality measure. This means that ASMRs can prove confusing, such that there is merit in adopting a standard population that is as representative of the actual population as possible. The most recent ESP (2013) was introduced to better reflect the contemporary population distribution of Europe, so this remains the most suitable ESP for the calculation of standardised rates; it must be noted, however, that this change may alter how we discuss the relative burden of CVD mortality across the continent and in some cases within countries.

## Conclusion

Age-standardised rates are commonly used in studying the epidemiology of a disease. Although changing the standard population will change the rates, this paper also shows that it may change the relative burden of disease when countries or subgroups are compared to each other, despite using the same mortality data. It is crucial that policy makers understand the effect of changes in standard populations on these comparisons. Similar effects as those found in CVD in Europe, due to the change in the European Standard Population, may be seen in other diseases that are also more prevalent in older age groups, such as cancer and dementia.

## Data Availability

The datasets generated and/or analysed during the current study are available in the European Detailed Mortality Database from the World Health Organization Regional Office for Europe repository: [https://gateway.euro.who.int/en/datasets/european-mortality-database/].

## Abbreviations

ASMR: Age-standardised mortality rates
ASMR13: Age-standardised mortality rates calculated using the 2013 European Standard Population
ASMR76: Age-standardised mortality rates calculated using the 1976 European Standard Population
CHD: Coronary heart disease
CVD: Cardiovascular disease
ESP: European Standard Population
ESP13: 2013 European Standard Population
ESP76: 1976 European Standard Population
ICD: International Classification of Disease
UK: United Kingdom
WHO: World Health Organization
